# Prevalence and Associations of Hypothyroidism in Indian Patients with Type 2 Diabetes Mellitus

**DOI:** 10.1155/2018/5386129

**Published:** 2018-08-09

**Authors:** Abilash Nair, C. Jayakumari, P. K. Jabbar, R. V. Jayakumar, Nishant Raizada, Anjana Gopi, Geena Susan George, T. P. Seena

**Affiliations:** ^1^Department of Endocrinology and Metabolism, Government Medical College, Thiruvananthapuram, India; ^2^Indian Institute of Diabetes, Thiruvananthapuram, India; ^3^Department of Endocrinology, University College of Medical Science, Delhi, India; ^4^Department of Pediatrics, ESIC Model Hospital Asramam, Kollam Kerala, India

## Abstract

Both type 2 diabetes and hypothyroidism are highly prevalent disorders in the community. The existing data regarding prevalence of hypothyroidism in patients with diabetes comes mostly from small studies. There are only two studies with a sample size of more than 1000 diabetic patients, none of which have been done in South Asians. The present study evaluated patients with type 2 diabetes for presence of hypothyroidism and the clinical factors associated with it. The demographic, anthropometric, clinical, and biochemical parameters of consecutively enrolled patients with diabetes were systematically collected and analyzed. A total of 1152 middle aged patients with type 2 diabetes with a mean duration of diabetes of around 10 years were enrolled. Nearly 40 percent of the patients were obese and overweight, respectively, for South Asian standards and abdominal obesity was seen in around 90% patients. Clinical hypothyroidism (TSH>10 mIU/ml) was present in 113 of patients (9.83%) and another 68 patients (5.9%) had subclinical hypothyroidism (TSH 5-10 mIU/ml). Anemia (odds ratio : 2.19), overweight/obese status (odds ratio 2.07), and known dyslipidemia (odds ratio : 1.99) were found to have independent association with clinical hypothyroidism. HbA1c, abdominal obesity, poor control of hypertension, lipid parameters, microalbuminuria, and renal dysfunction showed no difference among patients with hypothyroidism when compared with euthyroid patients. Subclinical hypothyroid patients had no difference in any of the above analyzed parameters when compared to the euthyroid patients. This study shows that a significant proportion of type 2 diabetes patients suffer from clinical or subclinical hypothyroidism and screening for the same may be appropriate.

## 1. Introduction

Type 2 diabetes and hypothyroidism are chronic diseases which frequently require lifelong follow-up and treatment. Both the diseases have long lasting effects on cardiovascular health and mortality with a higher risk attributable to the former [1, 2]. In addition to being a direct cardiovascular risk factor hypothyroidism contributes to cardiovascular morbidity by enhancing other risk factors like hyperlipidemia and hypertension [3]. Subclinical hypothyroidism of moderate severity is associated with higher risk of heart failure and stroke in the younger population [4]. Hypothyroidism has also been associated with nonalcoholic fatty liver disease, cancer mortality, arthritis, and kidney dysfunction but the causality in these situations is controversial [4]. A recent meta-analysis of 61 studies has shown a higher prevalence of subclinical hypothyroidism in patients with type 2 diabetes mellitus and higher prevalence of microvascular complications in the patients having both the conditions [5]. Both type 2 diabetes and hypothyroidism can be managed well in almost all patients to result in normalization of blood glucose levels and thyroid hormone levels which may reduce the morbidity of these conditions [6]. India is the diabetes capital of the world with the disease estimated to affect 6.5 to 19.5% of the adult Indians [7]. The situation is worse in the South Indian state of Kerala where the prevalence is the maximum among all Indian states [8]. The relation between diabetes and thyroid dysfunction has been studied by various researchers and the prevalence of hypothyroidism among patients with diabetes is reported from 4.8 to 31.4% [9]. Prevalence of hypothyroidism in India is around 11% [10]. South Indians have elevated thyroid-peroxidase antibody levels in around 9.5% of general population [11]. There is paucity of large studies from India which have looked into the prevalence of thyroid disorders in patients with diabetes mellitus. Through this retrospective study of diabetic outpatients visiting a tertiary care center, we estimated the prevalence of primary hypothyroidism and its association with other clinical parameters.

## 2. Materials and Methods

This study was conducted at Indian Institute of Diabetes, Thiruvananthapuram, which is a tertiary diabetes care center under the Government of Kerala. The hospital maintains an electronic record of the clinical, biochemical, and medication profile of all patients attending the hospital. The anthropometric measurements are done by the paramedical staff and the clinical details and medications are entered into the electronic records by the treating physicians themselves. Biochemical reports are entered into electronic records directly from the laboratory. This retrospective study was conducted by collecting clinical and biochemical data of Type 2 Diabetes patients attending the Institute. A dedicated staff was utilized to fill in a separate pro-forma for each patient by retrieving the records of individual patient. All type 2 diabetes mellitus patients attending the hospital and having a thyroid-stimulating hormone (TSH) test done during a one and a half year period from January 2016 were included in the study. Demographic parameters, duration of diabetes, anthropometric measurements, and blood pressure were noted from last visit records. History of addictions (smoking and alcoholism) and comorbidities (thyroid disease, hypertension, and dyslipidemia) was also noted from records. Laboratory data was collected from the patients' last visit details, including HbA1c, fasting plasma glucose (FPG), and 2-hour postprandial plasma glucose (2hr PPPG), lipid profile, serum renal function test, and hemogram. Thyroid-stimulating hormone (TSH) level estimated simultaneously with the above tests was noted. All the biochemical investigations were done in using the same equipment under a National Accreditation Board for Laboratories certified quality control program. Plasma glucose estimation was done with the glucose oxidase method (GOD-POD). Lipid parameters serum creatinine and uric acid were assessed using the autoanalyzer ERBA 360 (ERBA 360, India). HbA1c was estimated using a National Glucose Standardization Program certified HPLC system (BIORAD-D10, USA). Urine microalbumin was estimated in a nonfasting second voided urine sample with nephelometric method (MISPA I-2). Hemogram was estimated using Mindray-BC 3000 plus cell counter and ESR was estimated using the Westergren tube method. Thyroid-stimulating hormone (TSH) level was assessed using Eclysys 2000 Electrochemiluminescence system with commercially available kits (Roche, Germany). Each patient was classified as euthyroid (TSH 0.5-4.9 *μ*IU/ml), subclinical hypothyroid (TSH 5- 9.9 *μ*IU/ml), or clinical hypothyroid (TSH ≥ 10 *μ*IU/ml or previously on thyroxine replacement for hypothyroidism). Patients were labeled as hyperthyroid if they have been or are on antithyroid medications or have a TSH <0.5 *μ*IU/ml.

From the available parameters BMI and eGFR were calculated for each patient. The cardiovascular risk parameters like duration of diabetes, HbA1c, fasting and postprandial plasma glucose values, systolic and diastolic blood pressure, LDL cholesterol, eGFR, and other clinical parameters like hemoglobin levels and ESR were dichotomized based on appropriate clinical cut-offs. A waist hip ratio of > 0.85 and > 0.9 was considered to be having abdominal obesity in females and males, respectively. The prevalence was separately found out for known hypothyroidism, newly diagnosed clinical hypothyroidism, and newly diagnosed subclinical hypothyroidism.

For statistical analysis SPSS Statistics version 25 was used after tabulation into Microsoft Excel. Continuous variables are expressed as mean ± standard deviation, and categorical variables are expressed as frequencies and percentages. The variables were tested for their distribution and classified as normally distributed or not. Student's *t*-test and one-way ANOVA was used for continuous variables and chi square test and Fischer's exact test was used for categorical variables based on the distribution to compare groups. *P* value of less than 0.05 was considered significant.

## 3. Results

A total of 1152 patients were included in the study. The baseline clinical and biochemical parameters of these patients are shown in Tables 1(a) and 1(b). The patients had a mean age of 53 ± 11.15 years (mean ± SD). Most patients (82%) were native of Trivandrum District. The study sample consisted of 64.4% males. Around 7 and 16% of the subjects were smokers and alcohol users, respectively. The average duration of diabetes was 9.6 ± 7.8 yrs. The mean BMI was 26.7 ± 4.1 Kg/m^2^ and nearly 43% and 41% persons were obese and overweight, respectively. The average waist hip ratio was (0.98 ± 0.1). Abdominal obesity defined as a waist hip ratio of more than 0.85 for females and 0.9 for males was present in around 92 percent patients. Thirty percent patients had preexisting hypertension. The average systolic and diastolic BP were 131 ± 18 and 77 ± 8.8 mm of Hg, 58.2% persons had a systolic BP more than 130 mm of Hg, and 14.9 patients had a diastolic BP above 90 mm of Hg. Twenty-seven percent persons had known dyslipidemia. The mean HbA1c was 8.6 ± 0.3. The mean fasting and postprandial plasma glucose was 179.3 ± 2.3 mg/dl and 267.9 ± 111.9, respectively. Eighty-four and eighty-five percent had a poorly controlled fasting and 2 hr PP plasma glucose values, respectively, whereas 72% persons had a poorly controlled HbA1c value. The analysis of lipid parameters showed that 56% persons had a serum LDL level above the LDL target for diabetic persons (>100 mg/ dl). Nearly 30 percent of patients had microalbuminuria defined by a urine albumin: creatinine ratio of > 0.03 (30 mg/gram creatinine), 16.4% patients had an eGFR <60 ml/hr, and 40.6% persons had serum uric acid level above upper limit of normal. Apart from cardiometabolic risk factors, 26% patients had anemia (Hb< 12 g/ dl for females and Hb< 13 g/dlfor males); 33.9% persons had a raised ESR (>20 mm/hr).

### 3.1. Thyroid Dysfunction

8.33% patients had known history of hypothyroidism; 17 (1.5%) persons were diagnosed to have newly detected hypothyroidism (TSH>10 *μ*IU/ml) during the study period. Hence, the prevalence of clinical hypothyroidism was 9.83%. 68 (5.9%) patients were newly found to have subclinical hypothyroidism. When comparison was done between the clinical hypothyroid (preexisting and newly detected) and euthyroid persons (Tables 2(a) and 2(b)), the presence of hypothyroidism was found to be associated with female sex, hypertension, dyslipidemia, obesity, a duration of diabetes more than 2 years, anemia, and an elevated ESR. Alcohol intake was found to have a protective effect, but it could be due to a lower rate of alcoholism among females who were more likely to suffer from hypothyroidism due to other reasons. When alcoholism and ESR were adjusted for sex by logistic regression analysis no significant association of alcoholism and ESR with hypothyroidism remained. No difference was found in the prevalence of abdominal obesity, poor control of hypertension or lipid parameters, microalbuminuria, renal dysfunction, plasma glucose levels, HbA1c, or hyperuricemia among patients with hypothyroidism and those without. On logistic regression analysis, only 3 parameters,* namely, *known dyslipidemia (OR 1.99), overweight /obese status (OR 2.07), and anemia (OR 2.19), were found to be more common in patients with hypothyroidism. When subclinical hypothyroid patients were compared with the euthyroid group none of the variables analyzed showed a significant difference.

## 4. Discussion

In this study, patients with type 2 diabetes were assessed for presence of hypothyroidism based on the serum TSH levels. A comparison of clinical and biochemical features was then done among hypothyroid and euthyroid patients with diabetes. The majority of study population consisted of middle aged, overweight, male patients having diabetes for around a decade with moderately poor control of glycemic and lipid parameters. A large proportion (84%) of patients had overweight or obese status and nearly 90% persons had abdominal obesity defined by a high waist hip ratio. These findings are contrary to the popular belief that a majority of South Indian diabetic patients are lean. The study found a prevalence of clinical hypothyroidism in nearly 10% of patients with diabetes. Another 6% patients were found to have subclinical hypothyroidism.  Table 3 shows the prevalence of hypothyroidism reported in properly conducted previous studies with a sample size of at least 100 diabetic individuals. The data on the risk of thyroid dysfunction in type 2 diabetes is conflicting with many small studies showing a higher prevalence of hypothyroidism in patients with type 2 diabetes, whereas a large study from Norway showed a comparable prevalence of hypothyroidism in patients with or without type 2 diabetes mellitus [20]. Indian studies on this matter are few. A study from Imphal (India) done on 202 patients with type 2 diabetes mellitus noted a prevalence of 11.4% for hypothyroidism and 16.3% for subclinical hypothyroidism [15]. The prevalence was higher in elderly patients and with those higher BMI (>25 kg/m^2^) which is similar to the observation made in the current study. A small study from South India found prevalence of subclinical hypothyroidism to be present in 11.25% versus 7%, overt hypothyroidism in 12% versus 5%, and hyperthyroidism in 0.75% versus 1% in diabetic subjects versus controls [16]. The present study found a significantly high prevalence of hypothyroidism in females, which is a known fact among nondiabetic persons also. To our knowledge, the present study is the first to report an association of hypothyroidism with anemia/hemoglobin levels in patients with diabetes. Anemia is associated with hypothyroidism in nondiabetic subjects also. The reasons for association of anemia and hypothyroidism could be either due to the hypofunction of marrow, chronic disease, or a micronutrient deficiency leading to both for which the mechanisms need to be studied further. An association was also found to be present between duration of diabetes and hypothyroidism suggesting a contribution of cumulative effect of hyperglycemia in thyroid dysfunction although the current HbA1c levels did not correlate with the prevalence of hypothyroidism. Combined with the fact that previous studies have found a lower rate of autoimmunity in hypothyroidism occurring in diabetes, it could be hypothesized that thyroid dysfunction could be related to the microvascular or macrovascular dysfunction associated with long-standing diabetes. This view has been supported by a study by Hage et al. who found an association with microvascular complications of diabetes, respectively [21]. Although an association was found between hypothyroidism and high ESR the significance of the association was lost when results were adjusted for sex. Higher BMI and history of hyperlipidemia were significantly associated with presence of hypothyroidism. Whether treatment of hypothyroidism in diabetic patients leads to improvement in these cardiovascular risk factors needs to be evaluated.

Subclinical hypothyroid patients exhibited no difference in analyzed parameters when compared to euthyroid subjects. A recent meta-analysis by Han et al. has shown a 1.93 times higher prevalence of subclinical hypothyroidism in patients with type 2 diabetes mellitus [5]. They have also found increased risk of microvascular complications like for diabetic nephropathy (OR 1.74), diabetic retinopathy (OR 1.42), and peripheral neuropathy (OR 1.87) and macrovascular complication like peripheral arterial disease (OR 1.85) in the subclinically hypothyroid diabetic patients when compared to other diabetic patients. But another single large study has shown lower all-cause mortality in subclinically hypothyroid diabetic patients when compared to other diabetic patients [22]. In that study, cardiovascular mortality was also not increased in the subclinically hypothyroid patients. It may be hypothesized that in patients with diabetes the “normal” level of TSH may have an earlier age associated shift to right than the general population, whereby the mild increase in TSH levels is not accompanied by any adverse clinical consequences. Another possibility is that subclinical hypothyroidism may be a stage in the development of overt hypothyroidism without any clinical consequences during this intermediate stage although follow-up studies in a cohort of subclinical hypothyroid patients with diabetes need to be done to support or reject this hypothesis.

## 5. Conclusions

Clinical hypothyroidism is seen in around one-tenth of Indian patients with type 2 diabetes. Subclinical hypothyroidism is found in another 5 percent of patients. The presence of hypothyroidism in diabetic patients may be suggested by presence of female sex, hyperlipidemia, higher duration of diabetes, overweight or obese status, and presence of anemia. Subclinical hypothyroidism in diabetic population does not correlate with these factors. Whether the treatment of hypothyroidism improves cardiovascular outcomes and whether subclinical hypothyroidism has a potential to progress to clinical hypothyroidism needs to be studied.

## Figures and Tables

**Table:** (a) Demographic and biochemical features of patients studied (continuous variables)

**Variable**	**Mean (**±**SD)**
Age (years)	53.30 ± 11.2
Duration of Diabetes (years)	9.61 ± 7.8
Height (cm)	162.1 ± 9.1
Weight (kg)	70.2 ± 12.1
Body Mass Index (kg/m^2^)	26.7 ± 4.1
Systolic Blood pressure (mm of Hg)	131.0 ± 18.0
Diastolic Blood pressure (mm of Hg)	77.2 ± 8.8
Waist Hip ratio	0.98 ± 0.1
Hemoglobin (g/dl)	13.3 ± 1.7
Total Leucocyte count	6741.9 ± 2316
ESR (mm/hr)	18.4 ± 16.8
S. Creatinine (mg/dl)	1.0 ± 0.5
Urine Alb: Creat Ratio (mg/gram creatinine)	47.8 ± 77.7
TSH (*μ*U/L)	3.2 ± 7.3
AST (IU/ml)	31.7 ± 21.6
ALT (IU/ml)	40.3 ± 31.4
S. Cholesterol (mg/dl)	184.7 ± 44.5
S. Triglycerides (mg/dl)	135.2 ± 79.2
S. HDL (mg/dl)	46.3 ± 14.4
S. LDL (mg/dl)	111.2 ± 39.8
HbA1c (%)	8.6 ± 2.3
FPG (mg/dl)	179.3 ± 72.1
PPPG (mg/dl)	267.9 ± 111.9
S.Uric Acid (mg/dl)	4.8 ± 1.4

**Table:** (b) Demographic and biochemical features of patients studied (categorical variables)

**Variable**	Frequency (%)
Sex	
Male	742 (64.4)
Female	410 (35.6)
Smoker	84 (7.3)
Alcohol	183 (15.9)
Diabetes >2 yr	447 (38.8)
Known Hypertension	355 (30.8)
Uncontrolled systolic BP	670 (58.2)
Uncontrolled diastolic BP	172 (14.9)
Dyslipidemia	316 (27.5)
Known Hypothyroidism	96 (8.4)
Abdominal obesity	1059 (91.9)
Anemia	307 (26.6)
ESR>20 mm/hr	390 (33.9)
S. Creat> 1.2	167 (14.5)
Microalbuminuria	337 (29.3)
LDL > 70 mg/dl	970 (84.2)
LDL > 100mg/dl	650 (56.4)
HbA1C > 7%	831 (72.1)
FBS ≥ 110	964 (84.1)
PPBS ≥ 140	963 (85)
Clinical Hypothyroidism	113 (9.8)
All hypothyroidism including subclinical	181 (15.7)
Hyperuricemia	468 (40.6)
Overweight/obese	924 (83.9)
Obese	471 (42.8)
CKD	188 (16.4)

**Table:** (a) Comparison of clinical and biochemical parameters between clinical hypothyroid and euthyroid patients (continuous variables)

Variable	Clinical hypothyroid (n= 113)	Euthyroid (n= 971)	P value
	Mean ± SD	Mean ± SD	
Body mass Index (kg/m^2^)	28.20 ± 4.8	26.56 ± 3.9	**<0.001**
Age (yrs)	54.79 ± 9.9	53.14 ± 11.3	0.136
Duration of Diabetes (yrs)	10.5 ± 7.8	9.50 ± 7.8	0.211
Height (cm)	156.7 ± 8.5	162.64 ± 9.0	**<0.01**
Weight (kg)	68.85 ± 10.9	70.43 ± 12.3	0.14
Systolic BP (mm of Hg)	131.07 ± 16.7	130.97 ± 18.1	0.95
Diastolic BP (mm of Hg)	76.45 ± 8.2	77.24 ± 8.8	0.37
Waist Hip ratio	.98 ± 0.05	.98 ± 0.1	0.76
Hemoglobin (%)	12.2 ± 1.7	13.40 ± 1.6	**<0.001**
Total Leucocyte count	6570.54 ± 1648.4	6760 ± 2377.4	0.41
ESR (mm/hr)	23.41 ± 19.0	17.87 ± 16.4	**0.001**
Creatinine (mg/dl)	.95 ± 0.3	1.01 ± 0.5	0.23
Urine Alb: Creat Ratio (mg/g creatinine)	44.43 ± 73.0	48.14 ± 78.2	0.82
AST (IU/L)	32.13 ± 16.6	31.65 ± 22.1	0.82
ALT (IU/L)	38.75 ± 28.1	40.41 ± 31.7	0.59
S. Cholesterol (mg/dl)	180.42 ± 43.0	185.17 ± 44.6	0.28
S. Triglycerides (mg/dl)	127.81 ± 52.3	135.96 ± 81.5	0.30
HDL (mg/dl)	48.56 ± 13.2	46.08 ± 14.5	0.08
LDL (mg/dl)	106.30 ± 38.9	111.77 ± 39.9	0.165
HbA1c (%)	8.60 ± 2.0	8.62 ± 2.3	0.93
FBS (mg/dl)	171.71 ± 65.5	180.08 ± 72.7	0.24
PPBS (mg/dl)	258.51 ± 109.8	268.85 ± 112.1	0.35
Uric Acid (mg/dl)	4.71 ± 1.3	4.80 ± 1.4	0.53
eGFR (ml/hr)	83.86 ± 26.9	89.09 ± 46.4	0.24

**Table:** (b) Comparison of clinical and biochemical parameters between clinical hypothyroid and euthyroid patients (nominal variables)

Parameters	Hypothyroid (Clinical) (n=113)	Euthyroid (n=971)	P Value
	N (%)	N (%)	
Sex (M)	41 (36.2)	701 (67.5)	**<0.001**
Sex (F)	72 (63.7)	338 (32.5)	
Smoker	3 (2.6)	81 (7.8)	0.054
Alcohol	9 (7.9)	174 (16.7)	**0.014**
Duration > 2 yrs	80 (70.8)	625 (60.1)	**0.032**
Hypertension	48 (42.5)	307 (29.5)	**0.005**
Dyslipidemia	51 (45.1)	265 (25.5)	**<0.001**
Systolic BP> 130 mm of hg	68 (60.2)	602 (57.9)	0.58
Diastolic BP > 90 mm of hg	15 (13.3)	157 (15.1)	0.62
Abdominal obesity	107 (94.7)	952 (91.6)	0.22
Anemia	55 (48.7)	252 (24.2)	**<0.001**
ESR > 20 mm/hr	54 (47.8)	336 (32.3)	**0.002**
e-GFR <90 ml/min	16 (14.1)	172 (16.5)	0.49
Urine Alb: creat Ratio> 30mg/g	36 (31.9)	301 (29.0)	0.54
LDL > 100 mg/dl	59 (52.2)	591 (56.9)	0.34
HbA1C > 7 %	87 (77.0)	744 (71.6)	0.23
FBS > 110 mg%	93 (82.3)	871 (83.8)	0.68
PPBS > 140 mg%	95 (84.1)	868 (83.5)	0.89
Hyperuricemia	41 (36.3)	427 (41.1)	0.33
Overweight/obese	101 (89.4)	823 (79.2)	**0.015**
Obese	56 (49.6)	415 (39.9)	0.06

**Table 3:** Reviews on previous studies on prevalence of hypothyroidism in diabetes mellitus.

**First Author Year**	**Design, Sample size,** **Country**	**Prevalence of Clinical Hypothyroidism**	**Risk factors Identified if any**	**Remarks**
**Al-Geffari et al, 2013 [12]**	Retrospective, 411, Saudi Arabia	15.3%	F gender, duration of diabetes > 10 year	Nil
**Diez, 2011. [13]**	Cross Sectional, 318, Spain	3.5% ( overt hypothyroidism) 3.1 ( sub clinical)	Nil	No relation with duration of DM
**Palma, 2013 [14]**	Cross Sectional, 386, Brazil	0.7% (12.7 subclinical)	Nil	Nil
**Demitrost, 2012 [15]**	Retrospective, 202, Manipur, India	27.7	Age, BMI > 25	Nil
**Kumar RA 2017 [16]**	Retrospective, 400, Bangalore India	12%	Nil	Nil
**Akbar, 2006 [17]**	Case control, 100 diabetic patients with 100 controls, Saudi Arabia	16% (thyroid dysfunction)	Nil	Nil
**Perros, 1995 [18]**	Cross sectional, 1315	6.9% (Thyroid dysfunction in T2D)	Nil	Nil
**Celani, 1994 [19]**	Cross sectional, 290,	7.25%	Nil	Nil
**Fleiner, 2016 [20]**	Prospective, 1452, Norway		Nil	No significant relationship with T2DM

## Data Availability

The data used to support the findings of this study are available from the corresponding author upon request.
